# Sensitivity of *Aspergillus nidulans* to the Cellulose Synthase Inhibitor Dichlobenil: Insights from Wall-Related Genes’ Expression and Ultrastructural Hyphal Morphologies

**DOI:** 10.1371/journal.pone.0080038

**Published:** 2013-11-29

**Authors:** Gea Guerriero, Lucia Silvestrini, Michael Obersriebnig, Marco Salerno, Dietmar Pum, Joseph Strauss

**Affiliations:** 1 Department of Applied Genetics and Cell Biology, Fungal Genetics and Genomics Unit, University of Natural Resources and Life Sciences Vienna (BOKU), University and Research Center Campus Tulln-Technopol, Tulln/Donau, Austria; 2 Institute of Wood Science and Technology, University of Natural Resources and Life Sciences Vienna (BOKU), University and Research Center Campus Tulln-Technopol, Tulln/Donau, Austria; 3 Nanophysics Department, Istituto Italiano di Tecnologia, Genova, Italy; 4 Department of Nanobiotechnology, Institute for Biophysics, University of Natural Resources and Life Sciences Vienna (BOKU), Vienna, Austria; 5 Health and Environment Department, Austrian Institute of Technology GmbH - AIT, University and Research Center Campus Tulln-Technopol, Tulln/Donau, Austria; University of Wisconsin - Madison, United States of America

## Abstract

The fungal cell wall constitutes an important target for the development of antifungal drugs, because of its central role in morphogenesis, development and determination of fungal-specific molecular features. Fungal walls are characterized by a network of interconnected glycoproteins and polysaccharides, namely α-, β-glucans and chitin. Cell walls promptly and dynamically respond to environmental stimuli by a signaling mechanism, which triggers, among other responses, modulations in wall biosynthetic genes’ expression. Despite the absence of cellulose in the wall of the model filamentous fungus *Aspergillus nidulans*, we found in this study that fungal growth, spore germination and morphology are affected by the addition of the cellulose synthase inhibitor dichlobenil. Expression analysis of selected genes putatively involved in cell wall biosynthesis, carried out at different time points of drug exposure (*i.e.* 0, 1, 3, 6 and 24 h), revealed increased expression for the putative mixed linkage β-1,3;1,4 glucan synthase *celA* together with the β-1,3-glucan synthase *fksA* and the Rho-related GTPase *rhoA*. We also compared these data with the response to Congo Red, a known plant/fungal drug affecting both chitin and cellulose biosynthesis. The two drugs exerted different effects at the cell wall level, as shown by gene expression analysis and the ultrastructural features observed through atomic force microscopy and scanning electron microscopy. Although the concentration of dichlobenil required to affect growth of *A. nidulans* is approximately 10-fold higher than that required to inhibit plant cellulose biosynthesis, our work for the first time demonstrates that a cellulose biosynthesis inhibitor affects fungal growth, changes fungal morphology and expression of genes connected to fungal cell wall biosynthesis.

## Introduction

The fungal cell wall is a structure which plays a key role in coordinating cell growth and development. It can be schematically described as an intricate network of polysaccharides to which proteins are covalently or non-covalently associated [1]. It maintains fungal cell shape, contributes to osmoregulation, provides fungi with support and a physical barrier against mechanical stress and at the same time regulates processes like biofilm formation and adhesion to surfaces [1]–[2]. Fungal cell walls share a common “backbone architecture”, characterized by the occurrence of major structural polysaccharides, namely glucans, chitin/chitosan and mannans, associated with an amorphous matrix, made up of proteins and other polysaccharides [3]. Despite this common structure, the actual fungal wall composition is species-specific [4]. Fungal cell wall components have been shown to evolve faster than core metabolic genes [4], probably pushed by adaptive divergence to suit the broad variety of environmental niches that fungi colonize. Cell walls are indeed the outermost structures which are directly exposed to environmental constraints. Therefore, by responding to external stimuli and biotic/abiotic selective forces, they determine both fungal cell adaptation and the evolutionary success of a specific lineage [4]. Fungal walls are the result of the combined action of a set of core “housekeeping”-like genes, which are highly conserved among different fungal lineages, and a set of poorly conserved “accessory”-like genes [4]. Examples of “housekeeping”-like genes are those coding for wall biosynthetic enzymes (e.g. glycan synthases), while those encoding noncatalytic wall components (e.g. adhesins) belong to the repertoire of “accessory”-like genes [4].

The model filamentous fungus *Aspergillus nidulans* investigated in this work belongs to the Ascomycota phylum. Ascomycetous cell walls are bilayered, with a core containing load-bearing polysaccharides providing mechanical support to fungal cells and an outer layer of glycoproteins [1], [5]. The main polysaccharides in the wall of *A. nidulans* are β-1,3-, β-1,3;1,4- and β-1,6-glucans, chitin and α-1,3-glucans [6]–[8] and many of the genes involved in their biosynthesis have been functionally characterized [9]–[30]. Chitin synthase genes (*chs*) of fungal origin have been assigned to seven distinct groups, I to VII [26], [31]. This high diversity of *chs* is explained by their physiological roles, since different fungal *chs* have been shown to regulate several crucial developmental phases, as well as the formation of specific cellular structures [12]–[13], [17]–[18], [21], [24], [26], [32].

A recent genome-wide survey of cell wall-related genes in *A. nidulans* [1] has shown, among other cell wall-related genes, the occurrence of a putative β-1,3;1,4 glucan synthase, named *celA* (ANID_08444), together with a rich *chs* repertoire and one β-1,3 glucan synthase (*fksA*, ANID_03729) [33]. Functional characterizations of *celA* and *fksA* are still lacking in *A. nidulans*, however the occurrence of single genes (as opposed to the variety of *chs* existing in this model fungus) points to a particular, probably morphologically relevant role in wall biosynthesis.

Much attention has been traditionally paid and is still devoted to inhibitors specifically targeting the fungal cell wall, as they represent promising tools for the development of strategies to control the spread of threatening species [34]. As an example, one of the chief wall load-bearing polysaccharides, chitin, does not appear in the hosts of most fungal pathogens, therefore its underlying biosynthetic enzymes and pathways represent optimal targets for antifungals [35]. However, despite the great potential held by wall biosynthetic enzymes as target of potential antifungals, it is necessary to consider the high plasticity and dynamism shown by fungi in response to wall perturbing agents [36].

Several studies in literature have shown that exposure of filamentous fungi to sublethal concentrations of drugs specifically targeting the cell wall, such as for instance Congo Red (CR), Caspofungin, Echinocandin and Calcofluor White (CFW), can cause growth inhibition and morphological aberrant structures, together with the activation of the cell wall integrity (CWI) signaling pathway [37]–[38]. Cell wall inhibitors are a valuable tool to shed light on metabolic pathways regulating extracellular polysaccharide biogenesis and have been indeed used in algae [39]–[43], higher plants [44]–[52], oomycetes [53]–[58] and fungi [59]–[63].

Previous studies have also shown that dichlobenil (2,6-Dichlorobenzonitrile, DCB) can inhibit the synthesis of extracellular matrix (ECM) polysaccharides in the non-cellulosic red microalgae *Rhodella* and *Porphyridium* [39]–[40], which are important for their adhesion and motility and in the diatom *Achnanthes longipes* [41]. However, to the best of our knowledge, cellulose synthase inhibitors have not yet been tested in ascomycetous fungi as they have no cellulosic components in their cell wall, differently from cellulose-containing oomycetes.

Here we show for the first time that the cellulose synthase inhibitor DCB exerts inhibitory effects on *A. nidulans* growth and spore germination and triggers alterations in the morphology, topography and adhesive properties of hyphal surfaces. Moreover, to gain further insight into the fungal response to cell wall inhibitors, we compare the modifications triggered by DCB at the gene expression, morphological and ultrastructural level to those caused by CR. The results of this study start to shed some light onto the wall-related mechanism determining the response of *A. nidulans* to DCB, a herbicide classically used to selectively inhibit cellulose biosynthesis, and pave the way for future investigations on the presence of a similar response in other Ascomycetes, as well as in Basidiomycetes.

## Materials and Methods

### Fungal Cultivation

The fungal strains used in this study are listed in Table S1. The strains were grown on solid minimal medium (MM) supplemented with the indicated drugs (namely CR, DCB and CFW, alone or in combination). Media were prepared as described in [64], with 0.4% w/v glucose as carbon source and ammonium tartrate at final concentration of 10 mM as nitrogen source, and were supplemented with 0.02% trace metal solution [65].

CR was dissolved in water at the concentrations of 0, 10, 20, 50 and 100 µM, while DCB was prepared in 1% v/v methanol (MetOH) at the concentrations of 0, 40, 100 and 200 µM, as described in [57]. Consequently, the control condition for the DCB-treated mycelium was obtained by growing conidiospores in MM supplemented with 1% v/v final concentration of MetOH. CFW was used at the final concentration of 10 µg/mL.

Conidiospores’ suspension was obtained by gently rubbing the mycelium grown for 2 days on solidified MM with a sterile plastic spatula and approximately 1 mL of 0.01% v/v Tween-20. Conidiospores were used as inoculum to grow the mycelium in 20 mL liquid MM for 16 h on a rotary shaker at 180 rpm and 37°C. CR, DCB or 1% v/v MetOH were then added to the mycelium and the samples were harvested after 0, 1, 3, 6 and 24 h. The mycelium was collected using a layer of sterile Miracloth, dried using filter paper, immediately frozen in liquid nitrogen and either stored at −80°C or immediately processed for RNA extraction as described below.

### Spore Germination Test

In order to evaluate the effects of CR and DCB on the spore germination capability, the strain FGSC A4 was sporulated on solid MM not supplemented or supplemented with 100 µM CR and 200 µM DCB. After 2 days, spores were harvested and resuspended in 0.01% v/v Tween-20. For each condition, 10^2^ spores were counted under the light microscope (40×) by Fuchs-Rosenthal counting chamber, inoculated on solid MM and incubated at 37°C. After 2 days the developing colonies corresponding to germinated spores were counted and compared to the number of colonies obtained from the drug-less plates. The germination test was performed on three independent replicates.

### RNA Extraction and cDNA Synthesis

Total RNA from *A. nidulans* hyphae was extracted from 100 mg of finely pulverized tissue, by using the RNeasy Plant Mini Kit (Qiagen), coupled with the on-column DNaseI digestion. The quality of the extracted RNA was checked by electrophoresis and the concentration measured using a ND-1000 spectrophotometer (NanoDrop). One microgram of extracted RNA was retro-transcribed using the iScript cDNA Synthesis kit (Biorad), following the manufacturer’s instructions.

### Quantitative Real-time PCR

For quantitative real-time PCR analysis, approximately 20 ng cDNA were used as template. The cDNA was amplified using the iQ SYBR Green SuperMix (Biorad) on an iCycler IQ5 Real-Time PCR machine (Biorad). The reactions were performed in triplicate and repeated on two biological independent replicates. The PCR conditions consisted of an initial denaturation at 95°C for 10 min, followed by 40 cycles of denaturation at 95°C for 15 s, annealing/extension at 58°C for 60 s.

A dissociation kinetics analysis was performed at the end of the experiment to check the specificity of the products. PCR products were additionally checked with agarose gel electrophoresis and they all showed the presence of one band of the expected size. The primers used to perform real-time PCR analyses are reported in Table S2.

The results were analysed with the Q-gene software [66] and normalized using the housekeeping genes CRP2 and TEF1 (ID numbers ANID_05960 and ANID_02063, respectively), the last one already used for normalization in several fungal species [67]–[68].

A One Way ANOVA analysis was performed (with a Tukey test for pairwise multiple comparison procedures, as implemented in SigmaStat) and statistically significant (p<0.1) and very significant differences (p<0.05) are indicated in the graphs with one and two asterisks, respectively.

### Microscopic Analysis of A. nidulans Mycelia Grown in the Presence of CR or DCB

Conidia were germinated in liquid MM with 100 µM CR, 200 µM DCB or 1% v/v MetOH on coverslips in 60×15 mm Petri dishes overnight at 37°C. The following day the coverslips were removed from the plates using forceps, excess medium was removed using a Kimwipe, and the coverslips were mounted on glass slides and observed using an Eclipse E200 microscope (Nikon).

For confocal microscopy analysis, spores were grown on coverslips in MM supplemented with MetOH (1% v/v final concentration) or DCB (200 µM) for 1 day at 37°C. The following day, the grown hyphae were washed with fresh MM and incubated for 10 minutes in the dark with a solution containing 0.1% w/v of CFW (supplemented with 0.05% w/v of Evans Blue). The hyphae were then rinsed with fresh MM, mounted on glass slides and observed with an Olympus Fluoview FV1000 confocal microscope (excitation at 405 nm wavelength, emission between 426–479 nm wavelength).

### Protoplasting Efficiency Test

For the protoplasting efficiency test, condiospores were grown on a sterile cellophane sheet on solid MM supplemented with 1% v/v MetOH (control) or 200 µM DCB for 10–12 h at 30°C. The following day the cellophane sheets with the grown hyphae were removed using sterilized tweezers and were put in Petri capsules containing 50 mL filter-sterilized lysing solution (1% w/v *Trichoderma harzianum* lysing enzymes, Sigma L1412, in sterile KCl 0.7 M). The Petri capsules were incubated on a shaker at 50 rpm and 30°C for a total of 60 minutes. Formed protoplasts were counted using a Fuchs-Rosenthal counting chamber after 20, 40 and 60 minutes. The protoplasting efficiency was calculated by counting the formed protoplasts on a total of 50 cells for the control sample and on a total of 10 for the DCB-treated mycelium (the sporulation efficiency was severely affected in the presence of the drug).

### Atomic Force Microscopy

For atomic force microscopy (AFM) imaging, spores were grown overnight on coverslips, as described above, then mycelia were rinsed with sterile MM and water and were deposited on mica plates by using a cut-tip. Mild air-drying was carried out at room temperature (RT) before examination, by removing excess liquid from the sample surface (no blowing with nitrogen), as described in [69], but without the subsequent use of a Millipore filter to trap the cells. AFM measurements were taken immediately after drying, within a time-frame of maximum half an hour for each sample examination. Hyphal tips were imaged, as they constitute regions growing actively by apposition of new wall material.

The AFM images were taken on a Dimension Icon AFM (Bruker) using standard silicon probes (RTESPA or Tap300) by the same manufacturer and quantitative nanomechanical mode (QNM). In this mode the cantilever, which has a resonance frequency of ∼300 kHz, is dithered at 1 kHz, performing a small indent and collecting force-displacement data at each image point. These data are processed in real-time to provide maps of stiffness, adhesion, deformation and dissipation in addition to the usual topography. Images were processed using the software Gwyddion 2.28 (Czech Metrology Institute). The topography data were leveled by subtraction of the best fitting plane, and the color scales for all images of the same type (e.g. adhesion) were set such that the same color on different images corresponds to the same value of the represented quantity, which allows for a better comparison.

### Scanning Electron Microscopy

Fungal spores were incubated overnight at 37°C in 60×15 mm Petri dishes with liquid MM without and with 100 µM CR, 200 µM DCB or 1% (v/v) MetOH. The following day an aliquot of each sample was taken by sterile tip and spread on the surface of a double-sided adhesive conductive carbon disc and imaged in a low-vacuum scanning electron microscope (Inspect S50, FEI). Fungal spores and hyphae were investigated under low vacuum conditions (typically 0.65–0.80 mbar at 15.0 kV). Scans were recorded at magnifications in the range of 1,000x to 10,000x.

## Results and Discussion

### Effects of DCB on *A. nidulans* Growth

**Figure 1:**
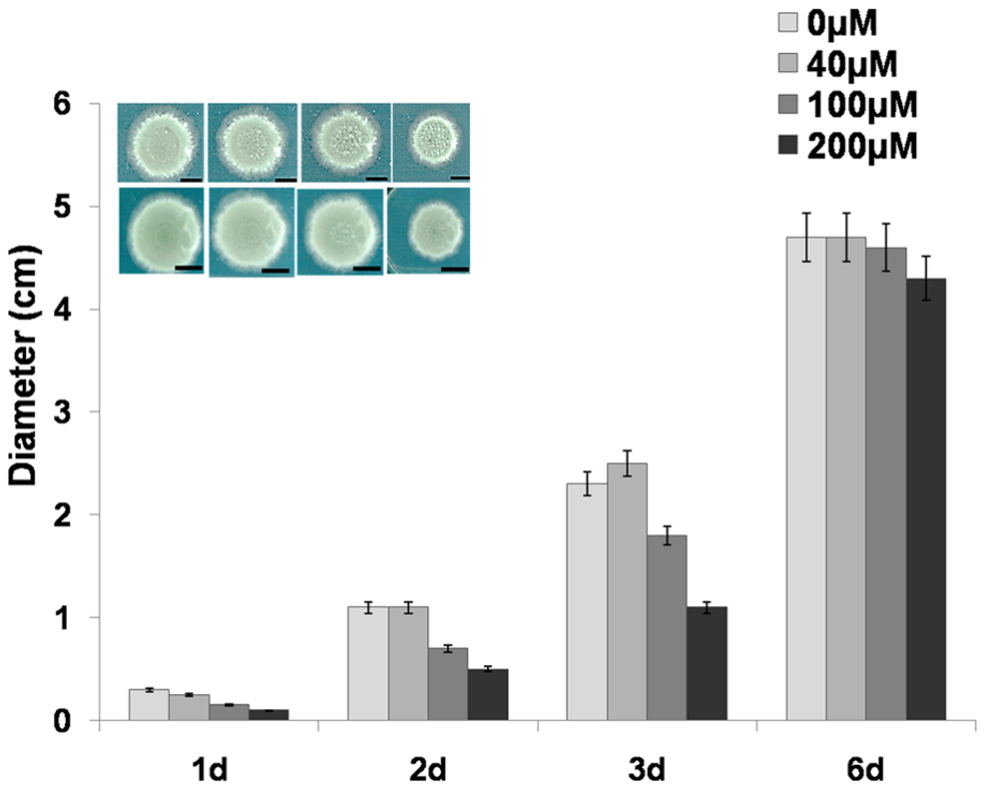
DCB decreases growth of *A. nidulans* mycelium. Growth of *A. nidulans* cultivated on solid MM supplemented with different concentrations of DCB, measured by taking the diameter of the mycelium over 6 days. Inset: images showing the mycelium grown for 2 and 3 days with increasing concentrations of DCB. Bars refer to 1 cm.

We performed a growth test by using different concentrations of DCB, namely 40, 100 and 200 µM. Growth was evaluated by measuring the colony diameter. As shown in Fig. 1, a growth inhibition could be observed at 100 and 200 µM. This effect was particularly evident at day 2 and 3. Indeed a smaller colony grew on solid MM supplied with 100 and 200 µM DCB (Fig. 1), while at 40 µM DCB no appreciable changes could be observed. At day 6 the growth differences were less evident than days 2 and 3: at longer incubation times *A. nidulans* might develop mechanisms which enable it to cope better with the drug. The adaptive response can be consequence of DCB metabolization, which has been documented in the literature to occur in animals and results in the formation of non-toxic glucuronides or estherified products [70]. Habituation of *A. nidulans* to DCB, as described for plant cells [71]–[73], might take place through a mechanism similar to the one described in plants. Plant habituation to DCB usually involves important changes in cell wall composition and in the metabolism of the cells: as an example, a decrease in cellulose and an increase in pectins has been shown in tomato cells habituated to the herbicide [71].

The inhibitory effect of DCB was observed for all the strains used (namely A4, SAA.111 and CS3007) and, interestingly, also for *A. niger* (MA169.4), although the effects were less pronounced with respect to *A. nidulans* (Table 1). These results suggest the existence of common response mechanisms to DCB in the genus Aspergillus and possibly in other Ascomycetes. The growth inhibitory effect in a non-cellulosic organism as *A. nidulans* is interesting, especially if one considers that concentrations of DCB within the range used in this study are required to decrease the growth of the cellulose-containing oomycete *Saprolegnia monoica* [57].

**Table 1:** Effect of DCB on the growth of the different *A. nidulans* strains used in this study and on *A. niger*.

	A4 MetOH	A4 DCB	SAA MetOH	SAA DCB	CS MetOH	CS DCB	*A. niger* MetOH	*A. niger* DCB
**1d**	0,87±0,06	0,77±0,06	1,07±0,06	0,87±0,06	1,10	0,90	0,87±0,06	0,87±0,06
**2d**	1,67±0,06	1,13±0,06	2,03±0,12	1,50	2,47±0,06	1,53±0,06	1,50	1,40
**3d**	2,60	1,73±0,12	3,03±0,12	2,27±0,06	3,37±0,06	2,17±0,06	2,07±0,06	1,70
**4d**	3,40±0,17	2,63±0,06	4,30	3,67±0,06	4,47±0,49	3,77±0,21	2,50	2,37±0,06
**6d**	3,07±0,12	2,57±0,12	4,20±0,17	3,53±0,21	4±0,10	3,83±0,06	2,97±0,15	2,80

Values are the means of 3 independent measures ± standard deviation.

DCB is a cellulose synthase inhibitor in higher plants [74], but it can also inhibit the biosynthesis of ECM polysaccharides in non-cellulosic organisms, as it was demonstrated in the red microalgae *Rhodella* and *Porphyridium* [39]–[40] and in the diatom *A. longipes* [41]. In particular, it was shown that DCB caused inhibition of protoplasts regeneration in the above-mentioned red algae, and that DCB-resistant mutants displayed higher amount of methyl galactosyl or xylosyl residues in their cell walls (in *Rhodella* and *Porphyridium*, [39]–[40]).

In higher plants DCB causes the accumulation of non-motile cellulose synthase (CESA) subunits, as demonstrated in *Arabidopsis thaliana* hypocotyls [75], most likely by interfering with the cross-talk between cytoskeleton and CESA complexes [76]. The DCB-cytoskeleton relationship was further supported by Rajangam et al. [49], who identified a microtubule associated protein from poplar (PttMAP20) as the target of the inhibitor. Moreover it was shown that DCB causes alteration of the cytoskeleton organization in *A. thaliana* [44]: low concentrations of this inhibitor caused disruption of the microtubular network, while high doses had effects also on the actin cytoskeleton. Interestingly in DCB-treated *A. thaliana* roots, pectin deposition was also significantly altered and Golgi bodies’ subcellular dynamics were blocked [44]. These findings indicate that the inhibitory action of DCB is broad and that its spectrum of action is not limited to cellulose deposition only.

In the diatom *A. longipes*, DCB (and its analogs) caused inhibition of ECM polysaccharides biosynthesis without interfering with the formation of the siliceous frustules [41]. The diatom uses ECM polysaccharides for initial attachment to the surfaces, for motility and for the synthesis of permanent adhesion structures [77]–[79]. It is known that cell wall components coordinate adhesion to organic/inorganic surfaces (e.g. [2]), which are crucial steps in establishing colonization in relevant pathogens, like *A. fumigatus* [80]–[82]. The results obtained in our study point to the existence of a DCB-responsive mechanism which triggers growth inhibition. Whether these mechanisms are similar to those acting in the non-cellulosic red algae and the diatom awaits further investigation.

### Formation of Aberrant Structures in *A. nidulans* and Inhibition of Spore Germination

**Figure 2:**
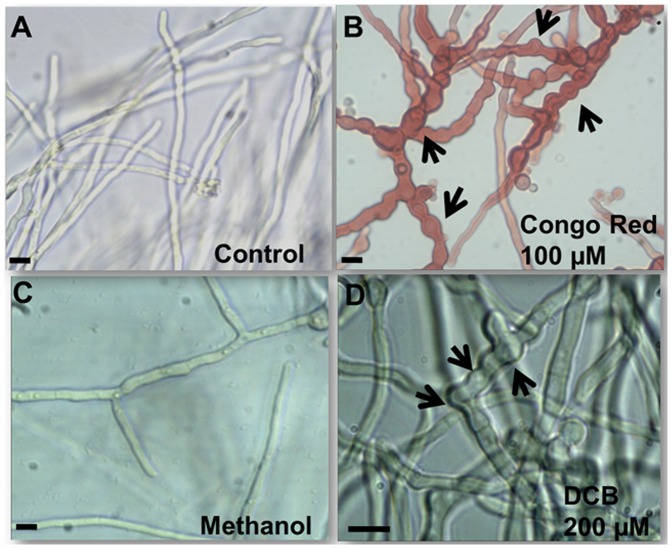
DCB and CR cause the formation of aberrant hyphal structures. Bright field microscopy pictures (40x in A, B and C, and 60x in D) of mycelium grown in liquid MM (A), in medium supplemented with 100 µM CR (B), 1% v/v MetOH (C) and 200 µM DCB (D). The arrows point to aberrant structures present along the hyphae (namely swollen regions and bulges). Scale bar refers to 10 µm.

Since DCB was found to inhibit *A. nidulans* mycelium growth, we decided to observe the morphology of the hyphae grown in the presence of the inhibitor. As can be seen in Fig. 2B, the hyphae grown in liquid MM supplemented with 200 µM DCB showed the presence of ripples and protrusions at the surface, in contrast to the control hyphae (grown in liquid MM with 1% v/v MetOH, Fig. 2A). The appearing ripples and protrusions suggest the occurrence of defects at the cell wall level. Moreover aberrant structures could also be observed along the hyphae of another *A. nidulans* strain studied, namely SAA.111: swollen and hypevacuolated germinating hyphae could be clearly observed at the microscope (Fig. S1B and inset therein).

The cell wall of *A. nidulans* responds to chemical perturbations and genetic manipulations by triggering the formation of anomalous structures. It has been shown, for instance, that deletion of *chs* genes can trigger the formation of “balloons” and intrahyphal hyphae [16]. These alterations of the fungal cell wall often result in modifications of its physico-chemical properties: for example deletion of genes involved in galactofuranose (Gal*f*) biosynthesis (*ugeA* and *ugmA*) induces different topographies of the hyphal surface, as revealed by AFM [83].

For the sake of comparison, we observed the response of *A. nidulans* to the well-known cell wall biosynthesis inhibitor CR [33]. As already previously reported [38], growth of the mycelium in the presence of this drug was reduced (Table 2) and this was accompanied by the occurrence of bulges (more frequent than in the DCB-condition) and swollen regions along the hyphae (Fig. 2D). The growth inhibition analysis was further extended: the effect of DCB was tested in combination with other known drugs acting at the cell wall level, namely CFW and CR. As can be seen in Table 2, the presence of DCB and CFW or DCB and CR in the medium enhanced the growth inhibitory effect triggered by the single drugs. These results therefore show the presence of a synergistic effect between DCB and other cell wall drugs and constitute an interesting starting point for further studies on antifungal agents.

**Table 2:** Effect of cell wall drugs alone or in combination with DCB on the different *A. nidulans* strains studied.

	A4 MM	A4 CR	A4 CFW	A4 DCB +CFW	A4 DCB +CR	SAA MM	SAA CR	SAA CFW	SAA DCB +CFW	SAA DCB +CR	CS MM	CS CR	CS CFW	CS DCB +CFW	CS DCB +CR
**1d**	0,83±0,06	0,77±0,06	0,70	0,70	0,60	1,13±0,06	0,97±0,06	0,77±0,12	0,93±0,12	0,70	1,00±0,10	0,93±0,06	0,83±0,06	0,87±0,06	0,77±0,06
**2d**	1,67±0,06	0,97±0,06	1,30±0,20	1,17±0,06	0,87±0,06	2,27±0,06	1,97±0,06	2,17±0,06	1,63±0,12	1,33±0,06	2,33±0,12	1,97±0,12	2,53±0,38	1,47±0,06	1,37±0,06
**3d**	2,57±0,06	1,37±0,12	1,87±0,15	1,63±0,06	1,30±0,10	3,23±0,12	2,77±0,15	2,73±0,06	2,13±0,06	2,27±0,06	3,23±0,15	2,90	3,03±0,15	1,93±0,06	2,20
**4d**	3,43±0,06	2,00±0,10	nd	nd	nd	4,37±0,12	3,80±0,26	nd	nd	nd	4,37±0,12	3,87±0,06	nd	nd	nd
**6d**	3,50±1,76	3,07±1,54	2,63±1,35	2,77±1,38	2,27±1,15	4,07±0,12	3,10±0,30	3,63±0,21	3,20±0,20	2,67±0,12	4,37±0,06	3,97±0,06	3,90±0,10	3,77±0,06	2,83±0,15

Values are the means of 3 independent measures ± standard deviation. nd stands for not determined.

**Figure 3:**
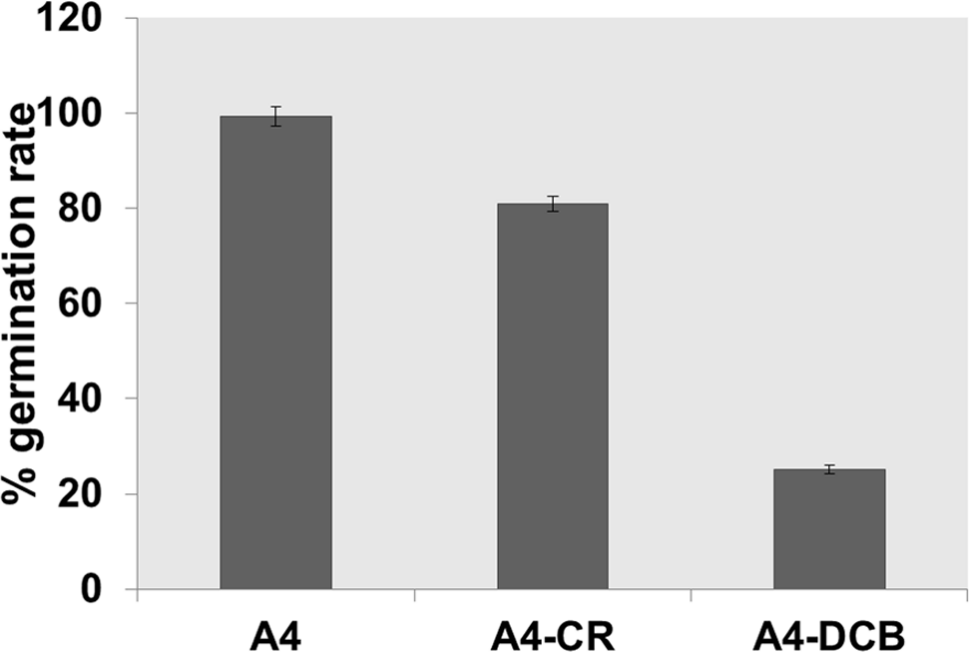
Germination of conidiospores in the presence of 100 µM CR and 200 µM DCB.

In order to understand whether DCB and CR could also affect the germination of conidiospores, we inoculated a defined number of spores (10^2^) in MM supplemented with 200 µM DCB and 100 µM CR. As can be seen in Fig. 3, both DCB and CR inhibited and/or delayed the germination of the conidiospores, but DCB exerted more dramatic effects with respect to CR, as addition of the drugs resulted in a reduction of germination of 25% and 80% respectively. The effect observed in the DCB-treated spores is more pronounced than the effect on the growth of mycelium, which can still grow, although at a slower rate (Fig. 1). This might be due to the different structure of spores’ cell walls, whose components are more loosely packed than in mycelial walls [84] and might therefore favour drug penetration.

The inhibitory and/or delaying effect of CR and DCB on conidiospores’ germination is also evident from SEM observations. In the controls, only the presence of hyphae was observed (fully germinated spores), while in CR- and DCB- treated samples, non-germinated spores were still present together with hyphae (Fig. S2).

### Cell Wall Genes’ Expression in CR and DCB-treated *A. nidulans*

**Figure 4:**
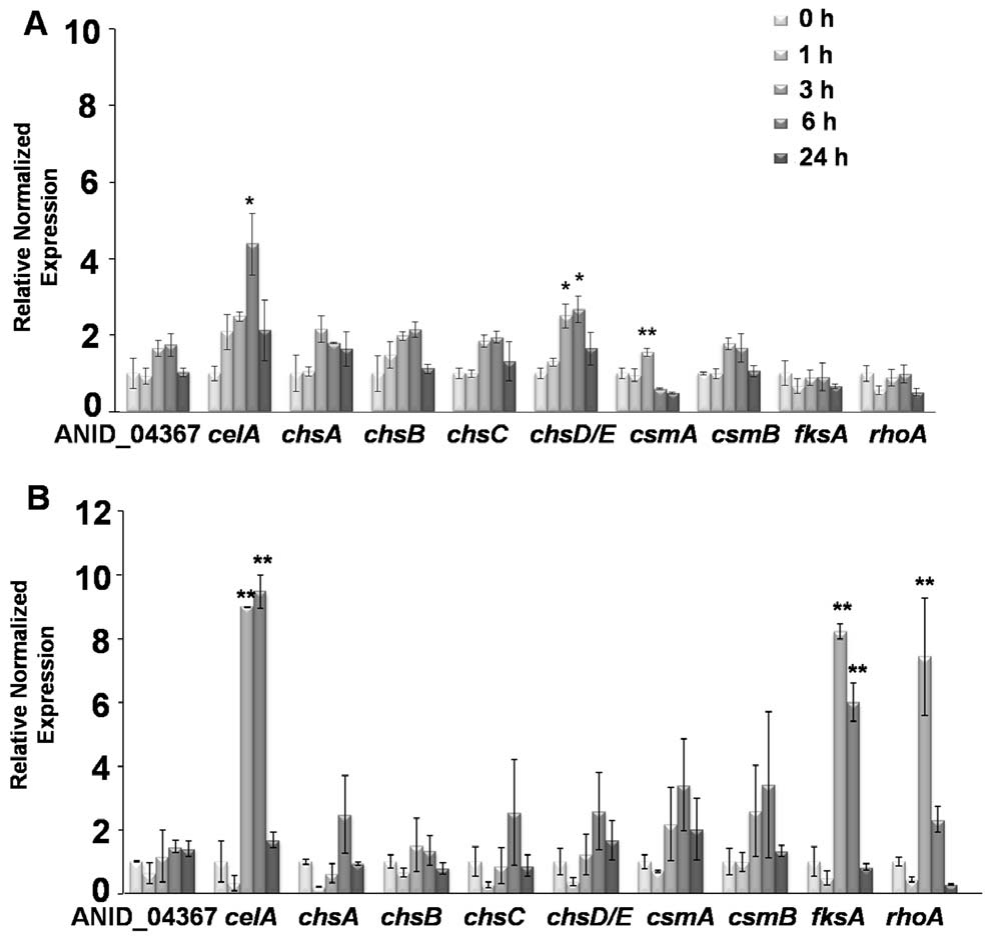
Gene expression analysis in CR and DCB-treated mycelium. Quantitative real-time PCR analysis of cell wall biosynthetic genes in *A. nidulans* grown in the presence of (A) 100 µM CR and (B) 200 µM DCB for 0, 1, 3, 6 and 24 h. Asterisks indicate significant (*) and very significant (**) changes.

Quantitative real-time PCR analysis carried out on *A. nidulans* grown in the presence of CR revealed an increase in *celA* and *chsD/E* expression after 6 h of drug exposure (p<0.1; Fig. 4A). After 24 h the mRNA steady-state levels of these genes decreased, most likely because of toxicity effects triggered by the drug (Fig. 4A). A mild (but significant, p<0.05) increase in *csmA* expression after 3 h was also observed (Fig. 4A). This might indicate a role in short-term signaling and in the transduction pathway which senses cell wall abnormalities, as already previously demonstrated for the myosin motor head domain-containing genes [27]. However, no statistically significant variations could be observed for the other *chs* studied. Likewise, neither the β-1,3 glucan synthase *fksA* nor the Rho-related GTPase *rhoA* showed significant variations in expression in response to CR (Fig. 4A).

In DCB-treated *A. nidulans*, the *chs* genes analysed did not show significant gene expression changes (Fig. 4B). The *celA* gene, however, strongly responded to the drug treatment already after 3 h, and with the highest steady-state mRNA levels observed after 6 h of exposure (Fig. 4B). Moreover, in contrast to what we observed for CR, *fksA* and *rhoA* showed a noteworthy increase in gene expression after 3 h of DCB treatment (Fig. 4B). These results indicate that *A. nidulans* responds to DCB by specifically modulating the expression of *celA*, *fksA* and *rhoA*.

The existence of a CWI transduction pathway has been demonstrated in *A. nidulans*, where two sensors belonging to the Wsc family (*wscA* and *wscB*) were shown to be involved in stress response [85]. Alterations at the cell wall levels are signaled through these sensors and result in growth adjustments in response to the stressor. Disruption of these genes affects cell wall composition by leading to an increase in the alkali-soluble cell wall glucans’ content, triggered by changes in the expression levels of the two α-1,3-glucan synthase genes *agsA* (whose expression decreased in the disruptants) and *agsB* (whose expression increased in the disruptants) [85]. In *A. nidulans* the downstream components of the CWI signaling pathway also determine the tolerance to antifungal drugs [85]: as an example, the dominant *rhoA* ^E40I^ strain has been shown to be hypersensitive to CFW and Caspofungin [86].

It is here worth highlighting that in our experimental set-up the relevant increase in *rhoA* expression suggests a role for the CWI signaling pathway in the response of the fungus to DCB. *A. nidulans rhoA* is indeed the ortholog of *Rho1* in *A. fumigatus* (94% identity and E-value of 2e-136 with BLASTP analysis), which was shown to be essential for CWI in this pathogenic fungus [87] and of *S. cerevisae Rho1* [88] (78% identity and E-value of 4e-106 with BLASTP analysis), which acts upstream of the CWI signaling pathway. Although further studies are necessary to confirm the actual role of the CWI signaling pathway in the response to DCB, our results open up interesting avenues for future studies.

### Confocal Microscopy Analysis and Protoplasting Efficiency of DCB-treated *A. nidulans*

**Figure 5:**
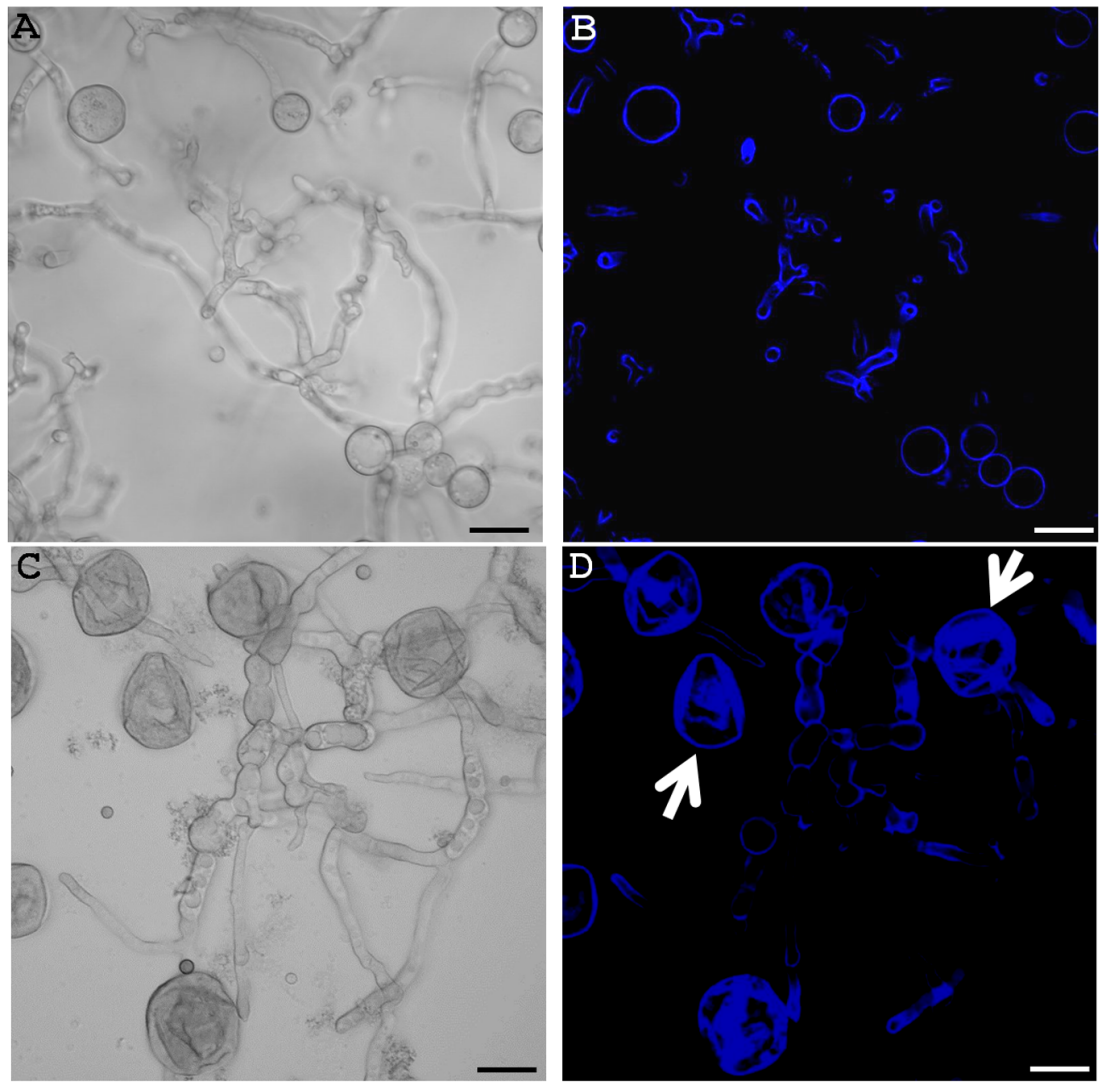
DCB does not affect chitin content and triggers the formation of wall “ghosts”. Confocal microscopy pictures of control (A and B) and DCB-treated *A. nidulans* (C and D). Differential Interference Contrast (DIC) images in (A) and (C) and CFW fluorescence images in (B) and (D). Arrows point to cell wall “ghosts”. Bars refer to 10 µm.

To shed light on the effects triggered by DCB, confocal microscopy analysis was performed on control and drug-treated mycelium stained with CFW. DCB clearly induced the formation of swollen and hypervacuolated hyphae (Fig. 5C) which are absent in the control (Fig. 5A). More importantly, the drug induced leaking of cytoplasmic content from the cells (arrows in Fig. S3), which then appear as cell wall “ghosts” (arrows in Fig. 5D). Fluorescence of these wall “ghosts” appears stronger (Fig. 5D), an effect most likely due to the curling/folding of the walls which have lost turgor pressure: the fluorescence along the hyphae and the cells that have not yet burst is indeed in the same range as the control (Figs. 5D and 5B, respectively). This result suggests that DCB does not induce an increase in chitin content, a finding which supports the gene expression analysis (Fig. 4B).

To further qualitatively assess the changes occurring at the cell wall after DCB exposure, protoplasting efficiencies were analysed. As can be seen in Table 3, a noteworthy decrease in protoplasting efficiency was observed in the DCB-treated sample with respect to its control. This finding suggests the presence of differences in the wall polysaccharides’ composition after drug exposure: it is here tempting to speculate that a higher content in mixed linkage β-1,3;1,4 and β-1,3 glucans resulting from the increased expression of *celA* and *fksA* (Fig. 4B) is the reason of the observed differences.

**Table 3:** Protoplasting efficiency (in %) of control and DCB-treated *A. nidulans*.

Time	A4 MetOH	A4 DCB
**0 min**	0%	0%
**20 min**	32%	0%
**40 min**	78%	10%
**60 min**	96%	0%

### Ultrastructural Morphological Differences in Drug-treated Hyphae

**Figure 6:**
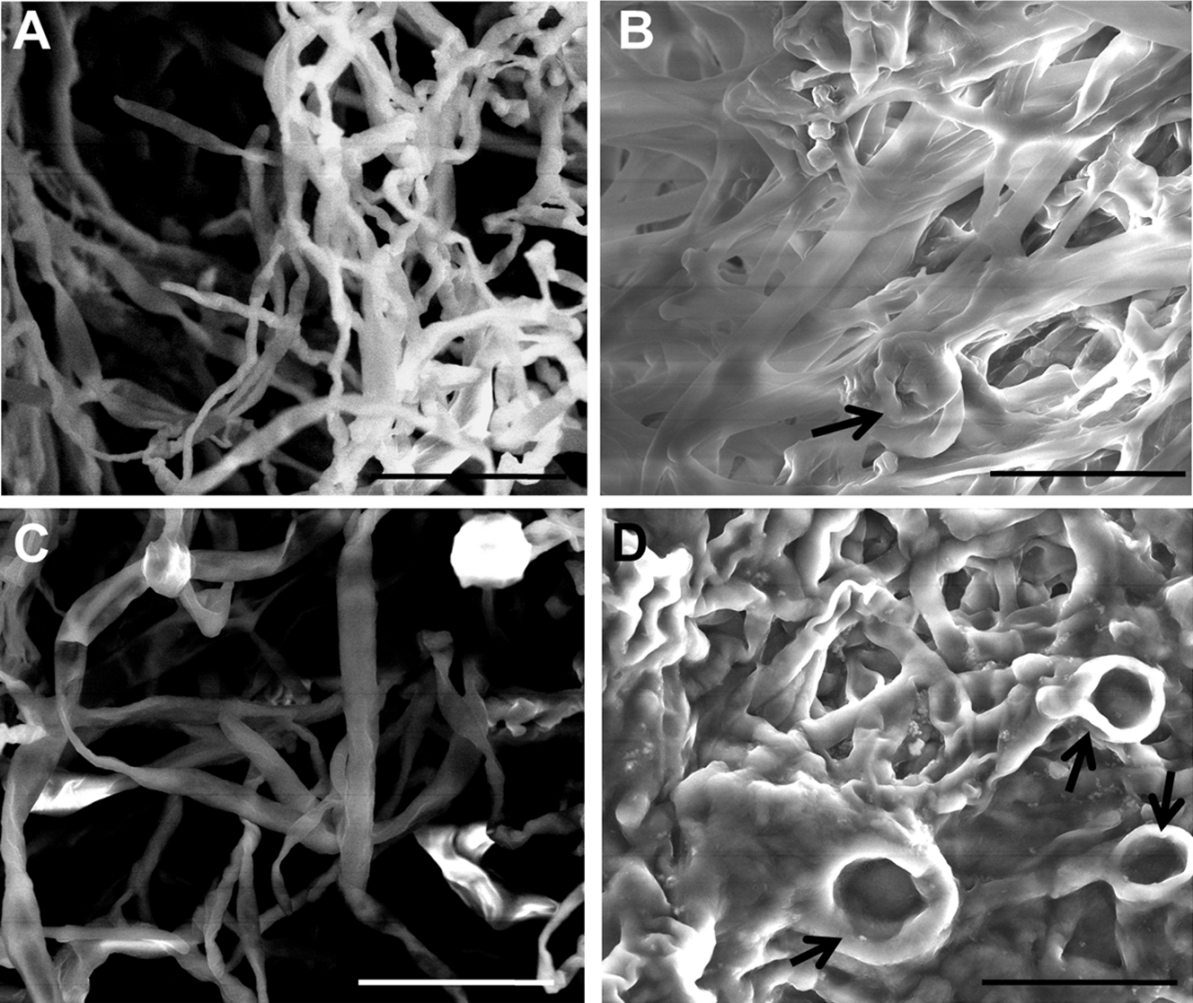
Hyphal ultrastructural features in the absence and presence of drugs. SEM pictures of (A) control, (B) CR-, (C) MetOH- and (D) DCB-treated hyphae. Arrows point to aberrant structures. Scale bar refers to 20 µm.

The morphological differences observed through optical microscopy were further validated by analysing control and drug-treated mycelia using SEM. The differences observed between control and treated *A. nidulans* at the SEM were striking (Fig. 6). CR- and DCB-treated hyphae were thicker than the respective controls and they appeared “glued”, without a clear separation of the single hyphal elements (Figs. 6B and 6D). Moreover, the presence of bulges were evident in the CR-treated hyphae (arrow in Fig. 6B), as already observed using optical microscopy (Fig. 2D). Several bulges could also be observed in the DCB-treated hyphae, which show annular structures, probably due to a local surface collapse triggered by the vacuum applied (arrows in Fig. 6D). These surface depressions could also indicate a weakened cell wall structure in the DCB-treated mycelium, which might therefore be more sensitive to the low vacuum applied during SEM imaging.

**Figure 7:**
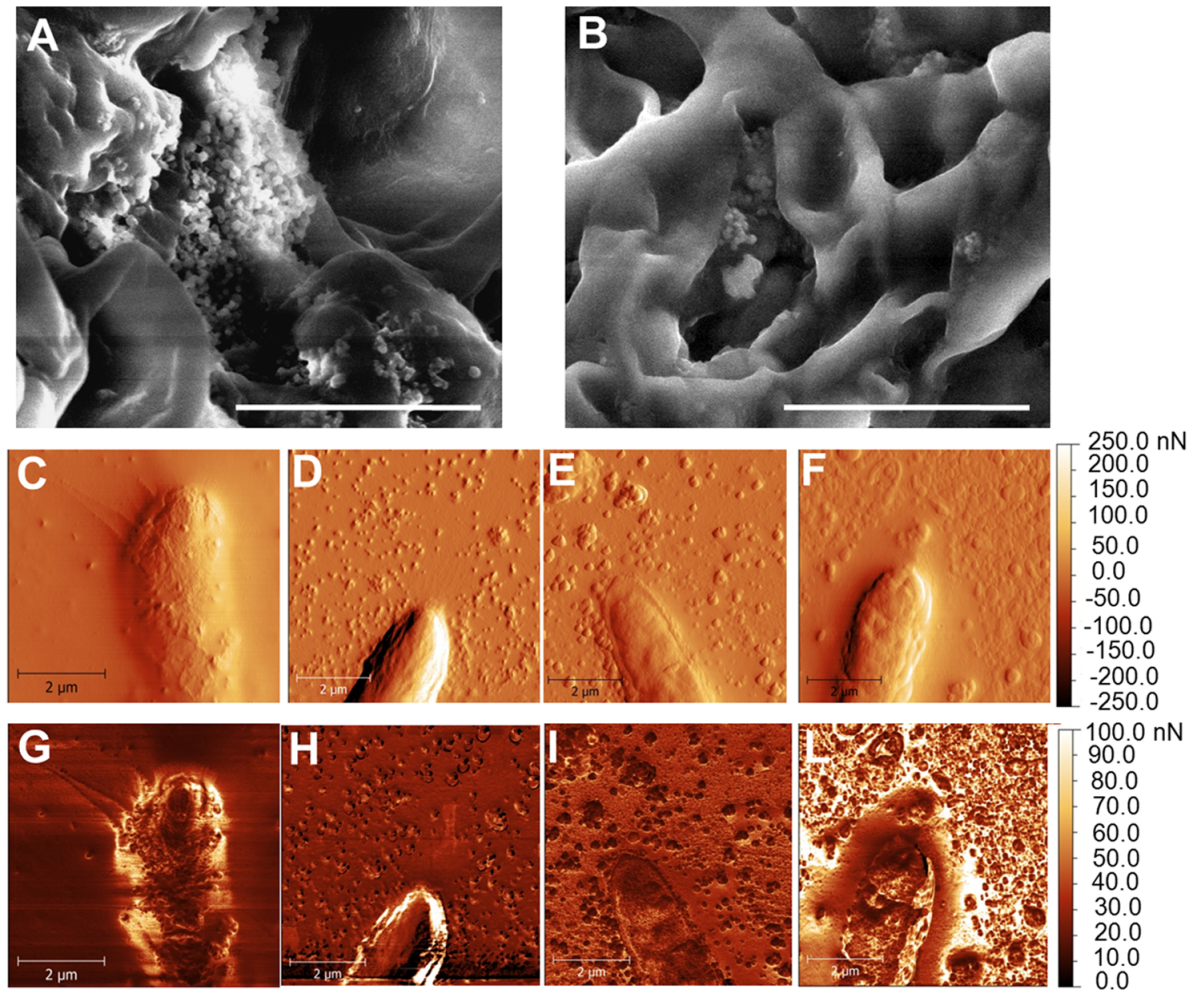
Hyphal ultrastructural differences triggered by CR and DCB. (A–B): SEM pictures of (A) CR- and (B) DCB-treated mycelium, showing the presence of small particles accumulating on the hyphal surface. (C–L): AFM images, showing surface topographical gradient (C–F) and adhesion maps (G–L) obtained on samples grown overnight at 37°C in control condition (C and G), with 100 µM CR (D and H), with 1% v/v MetOH (E and I) and 200 µM DCB (F and L). Bars refer to 10 µm (A and B) and 2 µm (C–L).

As a further means to assess the ultrastructural differences triggered by the drugs, AFM was used to obtain both the topographies and the adhesion maps of hyphal surfaces. AFM has already shown strong potential for fungal cell wall analysis [89] and has provided real breakthroughs in the field (e.g. [90]). Both SEM and AFM revealed the presence of many extracellular particles appearing after drug treatment with CR and DCB (see Figs. 7A and 7B for SEM, and Figs. 7D and 7F for AFM, respectively). On the contrary, the AFM image of the control (Fig. 7C) showed only very few particles. However, AFM revealed the presence of similar particles also in the MetOH-treated sample (Fig. 7E). Since we observed leaking of cytoplasmic content at the confocal microscope (Figs. 5C and 5D) and since tip bursting is a phenomenon which has been reported in oomycetes treated with wall biosynthesis competitive inhibitors (e.g. [91]), it is tempting to speculate that the granular material here observed might be a consequence of intracellular material release from the hyphae, or could come from partial wall degradation, following the stress levels experienced. Interestingly, we observed at the AFM the presence of hyphae lacking part of the outer surface layer after exposure to DCB and unmasking a rougher surface (Fig. S4B), a feature neither observed in the respective control (Figure S3A) nor in the other conditions studied (not shown). Since the samples were all processed at the same time and in the same way, we exclude the possibility of surface damage in the DCB sample triggered by the technical preparation before AFM.

The particles observed in the AFM images of MetOH- and particularly DCB-treated samples (Figs. 7E and 7F, respectively) present on the average a larger size than those observed in the CR-treated sample (Fig. 7D), which could be an effect of particle aggregation occurring after their release on the surrounding substrate. Additionally, when examining the top surface of the hyphal region, a highest local roughness appears on the DCB-treated sample (Fig. 7F), in agreement with the previous observation of outer layer removal following exposure to the drug (Fig. S4).

Since the AFM is basically a force sensor, during the same measurements as the topographical images shown previously it was also possible to measure the adhesion force between probe tip and sample surface, and the respective maps are shown in Figs. 7G–7L. Similarly to the topographic images, again in the adhesion maps the presence of particles and particle aggregates on the surrounding substrate is clear in Figs. 7H–7L. The DCB-treated sample shows the most corrugated pattern also in adhesion (Fig. 7L), corresponding to domains separated by alternated low and high adhesion values. The adhesion outside the hyphae is also the highest in the DCB-treated sample, as compared to CR- and MetOH-treated samples, (in the former, Fig. 7H, in particular, the hyphal edges exhibit most contrast with respect to Fig. 7I, which is probably a topographical artifact). The control sample (Fig. 7G), on the contrary, shows low adhesion both on top of the hyphae and on the surrounding background, again with peak values at the edges only (topographic artifact).

**Figure 8:**
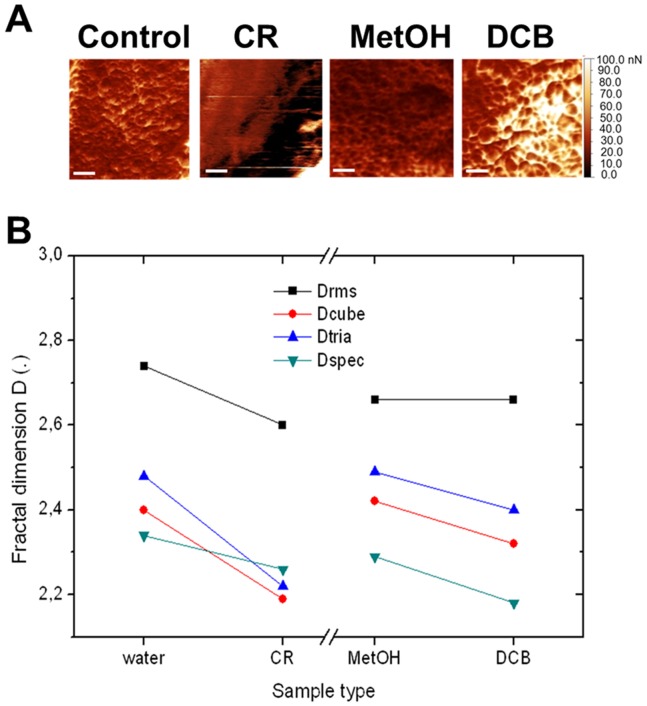
Fractal analysis of AFM images. (A) Close-up of the adhesion maps in Fig. 6 on the hyphal surfaces. Scale bars refer to 0.5 µm. (B) Plot representing the fractal dimension D obtained from the images in (A) according to different calculation tools available in Gwyddion, namely: Drms from root mean square of adhesion values, Dcube and Dtria from differently shaped (cube or tetragonal) boxes used for coverage of the image surface, and Dspec from Fourier spectrum of the spatial frequencies contained in the image.

To quantitatively characterize the patterns appearing on the hyphal surface, we performed a fractal analysis of the hyphal regions of the adhesion images (Fig. 8). Since its introduction [92], fractal geometry has been widely used to describe complex natural phenomena, as it allows for a description of natural objects’ geometry that is deeper than that of traditional Euclidean geometry. From fractal analysis a parameter called fractal dimension D can be extracted, which for a 2D surface is higher than the 2 representing a flat surface, and lower than the 3 representing the most complex convolution of the surface, completely filling the 3D volume of its space. Thus, roughly speaking, D describes the complexity of the object texture. Whereas fractal analysis is usually applied to topographical AFM or profilometer features (see e.g. [93]–[95]), no fundamental reason prevents one from applying it also to different types of surface images. For example, it has been applied on SEM images in [96], and on AFM images of adhesion, similar to the present case, in [97]. In particular, in our samples, the adhesion maps may represent the values of surface energy resulting from the treatment of the hyphae with the respective drugs, which would finally allow to assess their effect on the change of the respective physical property.

From the adhesion maps reported in Fig. 8A, the values of D according to the different calculation tools available to this goal in Gwyddion have been plotted in Fig. 8B. Independent on the absolute D values (with overall range of 2.1–2.7), for all calculation methods a decrease in D is observed when moving away from the respective control image (water for CR and MetOH for DCB). This decrease in complexity of the surface texture on administration of the drug can be interpreted as a re-arrangement of the cell wall physical properties, in particular in the direction of a disorganization of its subunits present in the control hyphae. This observation is in agreement with the simultaneous appearance of the particulate matter on the surrounding substrate, as previously commented.

Overall these data indicate that both CR and DCB alter the physical properties of the cell wall. Altered cell wall structures can indeed result in different physical properties, as demonstrated in the *A. nidulans* defective in Gal*f* biosynthetic genes [83]: these strains display loosely packed cell wall surface subunits which expose polar groups normally masked. These results are also in agreement with the role of Gal*f* in reducing adhesion in *A. fumigatus*, whose absence in the cell walls unmasks a mannan layer which in its turn increases adhesion to inert and biological surfaces [98]. It is hence plausible to assume that the treatment with cell wall drugs induces alterations in wall architecture which translate into an alteration of wall biosynthetic genes’ expression, remodeling of the wall architecture, modified morphologies, topographies and adhesion properties.

## Conclusions

In this study gene expression and microscopy analyses on the effects triggered by the inhibitors CR and DCB on cell wall were carried out in the model filamentous fungus *A. nidulans*. For the first time an inhibitory effect of DCB on growth and spore germination is reported for a filamentous fungus. Our results indicate indeed that, despite being a non-cellulosic organism, *A. nidulans* shows a response to the drug, and this suggests the presence of DCB-responsive gene(s) regulating fungal growth. It remains to be elucidated how DCB exerts its action: future studies can explain whether the mechanism of action is similar to that operating in plants and whether the fungal cytoskeleton is affected by the drug exposure. On a longer perspective, our study paves the way for future investigations on other fungal species, including clinically-relevant ones.

## Supporting Information

Figure S1 DCB triggers the formation of swollen and hypervacuolated hyphae in SAA.111. Bright field microscopy pictures (60x) of SAA.111 grown in liquid medium supplemented with 1% v/v MetOH (A) and 200 µM DCB (B). Insets: germinating hyphae. Scale bar refers to 10 µm.
(TIF)

Figure S2 CR affects *A. nidulans* spores’ germination. SEM image showing the presence of non- germinated conidiospores in CR-treated *A. nidulans*. Both hyphal bulges and conidiospores show depression and invaginations on the surface caused by the vacuum applied. Scale bar refers to 20 µm.
(TIF)

Figure S3 DCB causes leakage of cytoplasmic content. Confocal microscopy pictures of DCB-treated *A. nidulans* in DIC (A) and CFW fluorescence (B). Arrows point to cytoplasmic content leaking out from cells. Bars refer to 10 µm.
(TIF)

Figure S4 DCB causes peeling off of the hyphal outer surface layer. AFM image (topographical gradient) showing surface detail of MetOH-treated hyphae (A) and image showing peeling off of the outer surface layer (arrows) in DCB-treated *A. nidulans* (B).
(TIF)

Table S1 List of the fungal strains used in this study.
(DOC)

Table S2 Gene symbol, name and sequences of primers used for quantitative real-time PCR.
(DOC)
