# Supplementary figures and images for: Sensitivity of Aspergillus nidulans to the Cellulose Synthase Inhibitor Dichlobenil: Insights from Wall-Related Genes’ Expression and Ultrastructural Hyphal Morphologies
Source: PLoS One. 2013 Nov 29;8(11):e80038. doi: 10.1371/journal.pone.0080038 (PMC3843659; doi:10.1371/journal.pone.0080038)

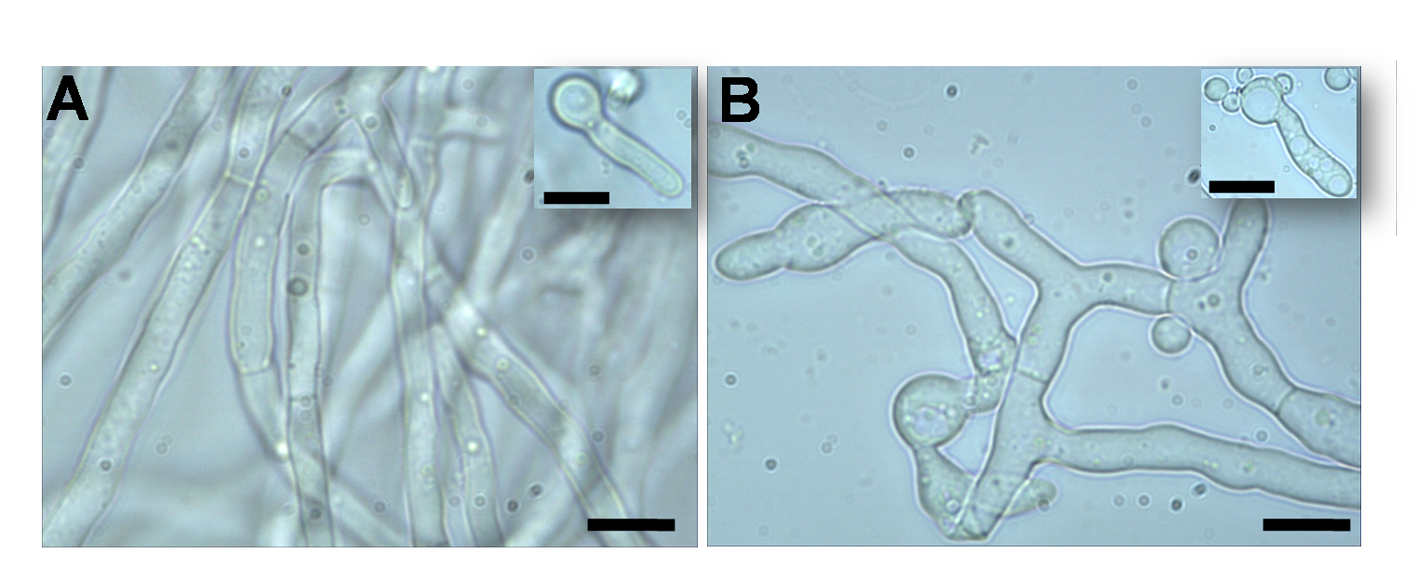

Supplement: Figure S1 — DCB triggers the formation of swollen and hypervacuolated hyphae in SAA.111. Bright field microscopy pictures (60x) of SAA.111 grown in liquid medium supplemented with 1% v/v MetOH (A) and 200 µM DCB (B). Insets: germinating hyphae. Scale bar refers to 10 µm. (TIF) [file pone.0080038.s001.tif]

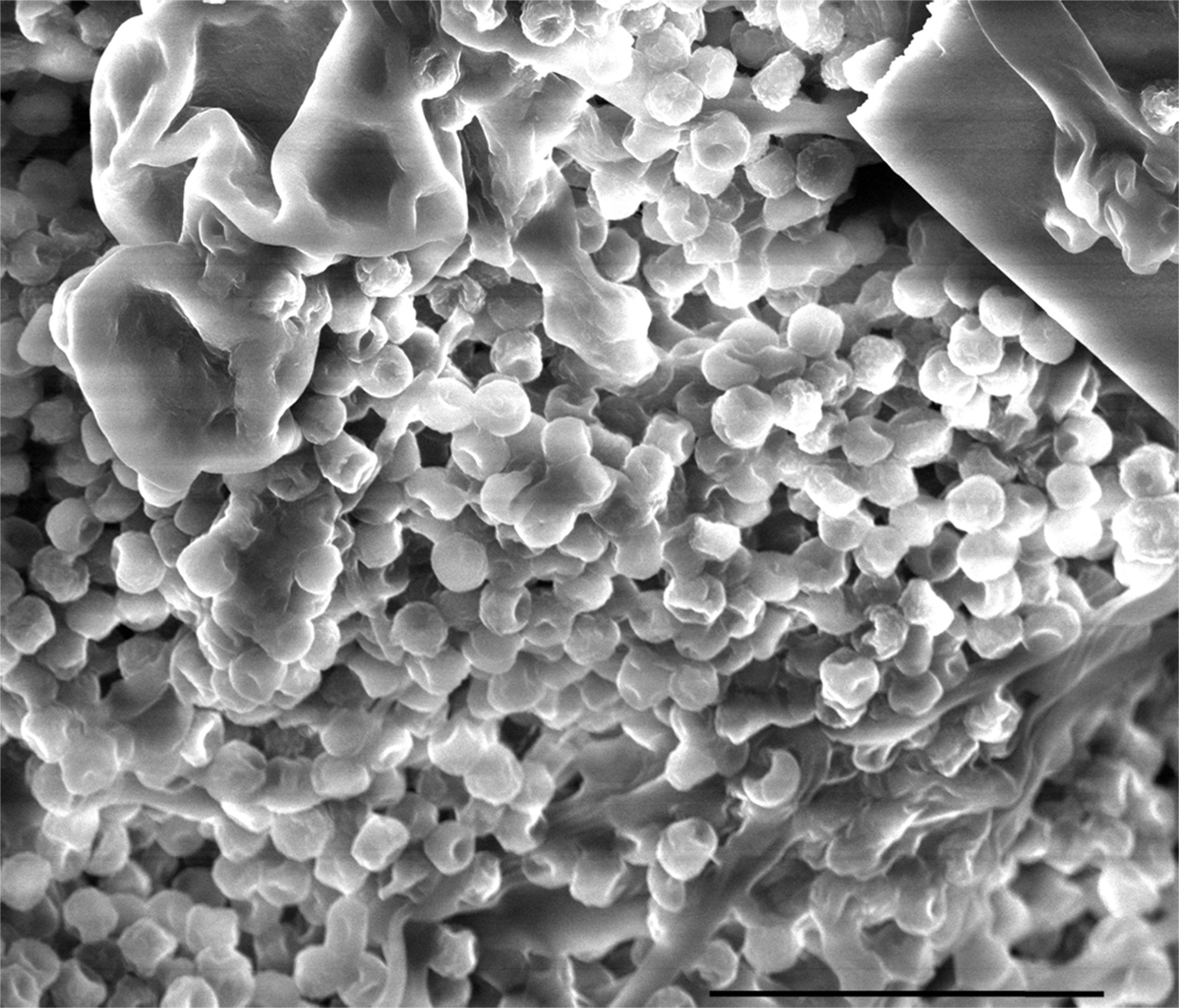

Supplement: Figure S2 — CR affects A. nidulans spores’ germination. SEM image showing the presence of non- germinated conidiospores in CR-treated A. nidulans. Both hyphal bulges and conidiospores show depression and invaginations on the surface caused by the vacuum applied. Scale bar refers to 20 µm. (TIF) [file pone.0080038.s002.tif]

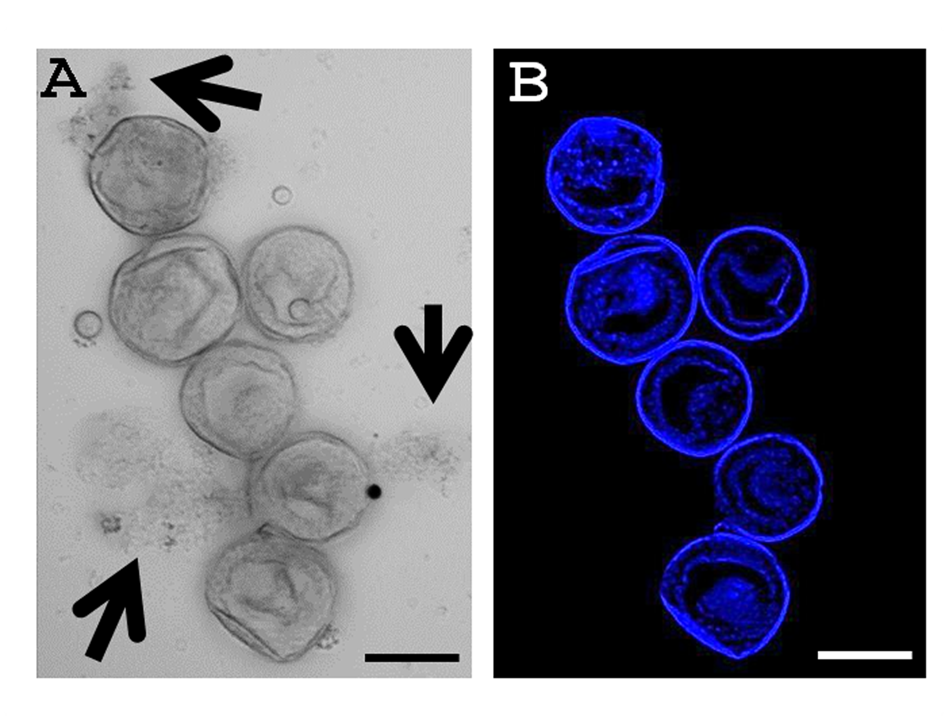

Supplement: Figure S3 — DCB causes leakage of cytoplasmic content. Confocal microscopy pictures of DCB-treated A. nidulans in DIC (A) and CFW fluorescence (B). Arrows point to cytoplasmic content leaking out from cells. Bars refer to 10 µm. (TIF) [file pone.0080038.s003.tif]

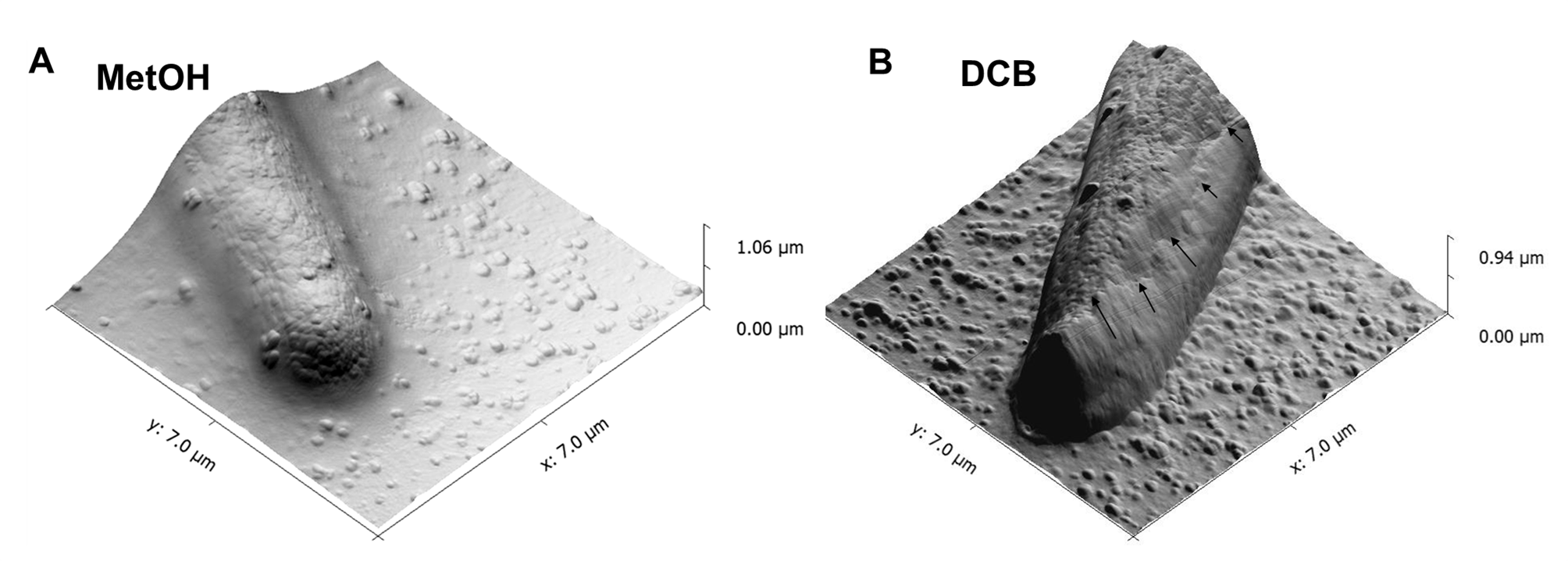

Supplement: Figure S4 — DCB causes peeling off of the hyphal outer surface layer. AFM image (topographical gradient) showing surface detail of MetOH-treated hyphae (A) and image showing peeling off of the outer surface layer (arrows) in DCB-treated A. nidulans (B). (TIF) [file pone.0080038.s004.tif]
