# Supplementary material for: Sensitivity of Aspergillus nidulans to the Cellulose Synthase Inhibitor Dichlobenil: Insights from Wall-Related Genes’ Expression and Ultrastructural Hyphal Morphologies
Source: PLoS One. 2013 Nov 29;8(11):e80038. doi: 10.1371/journal.pone.0080038 (PMC3843659; doi:10.1371/journal.pone.0080038)
Supplement: Table S1 — List of the fungal strains used in this study. (DOC) [file pone.0080038.s005.doc]

| **Strain abbreviation used in this study** | **Full name** |  | **Genotype** |  |  |  |
| --- | --- | --- | --- | --- | --- | --- |
| A4 | FGSC A4 (Glasgow wild-type) |  | *veA+* |  |  |  |
| SAA | SAA.111 |  | *veA1; biA1;* Δ *argB :: trpC; riboB2; pyroA4; wA3* |  |  |  |
| CS | CS3007 |  | *biA1; argB2* |  |  |  |
| *A. niger* | *A. niger* (MA169.4) |  | *KusA::amdS; pyrG* |  |  |  |
