# Supplementary material for: Sensitivity of Aspergillus nidulans to the Cellulose Synthase Inhibitor Dichlobenil: Insights from Wall-Related Genes’ Expression and Ultrastructural Hyphal Morphologies
Source: PLoS One. 2013 Nov 29;8(11):e80038. doi: 10.1371/journal.pone.0080038 (PMC3843659; doi:10.1371/journal.pone.0080038)
Supplement: Table S2 — Gene symbol, name and sequences of primers used for quantitative real-time PCR. (DOC) [file pone.0080038.s006.doc]

| **Gene (gene symbol)** | **Primer name** | **Sequence (5’ → 3’)** |
| --- | --- | --- |
| ***celA* (ANID_08444)** | **celAqPCRFwd** | ATGGGAGATTCTTGCGACTG |
|  | **celAqPCRRev** | TCCTGTGAGTGTTCGCTTTG |
| ***fksA* (ANID_03729)** | **FksqPCRFwd** | ATCCATCTGGGTGCTTTCTG |
|  | **FksqPCRRev** | ACCAAAACCAACAGCAGACC |
| ***rhoA* (ANID_05740)** | **rhoqPCRFwd** | ATTTGCTTCGCTGTCGACTC |
|  | **rhoqPCRRev** | GCGAAGATCCTTCTTGCATC |
| ***chsC* (ANID_04566)** | **chsCqPCRFwd** | GCAAAGATGCCTGGAAGAAG |
|  | **chsCqPCRRev** | TAACGTCTTTCCCGTTGACC |
| ***chsA* (ANID_07032)** | **chsAqPCRFwd** | AGGACGAAACCCACTTTACG |
|  | **chsAqPCRRev** | ATCTTTGCCCCATGTACGAG |
| ***chsB* (ANID_02523)** | **chsBqPCRFwd** | CGACAAGGACACATTGGATG |
|  | **chsBqPCRRev** | TGAGTTGCTGGTTTGGAGTG |
| **ANID_04367 (Class III *chs*)** | **AN4367qPCRFwd** | TTTCCGCCTATCGTTACAGG |
|  | **AN4367qPCRRev** | CCCTTCAATCCCCTTCTTTC |
| ***chsD/E* (ANID_01555)** | **chsD/EqPCRFwd** | TCGTCATCTTCGTTGAGCTG |
|  | **chsD/EqPCRRev** | CAAGAATAAGGGCGAGCAAG |
| ***csmA* (ANID_06318)** | **csmAqPCRFwd** | TGCCAGACTCAGCATTGAAG |
|  | **csmAqPCRRev** | ACATTGCCGTCCTTACCAAG |
| ***csmB* (ANID_06317)** | **csmBqPCRFwd** | TGCCTTATGTGGTCGTTGTC |
|  | **csmBqPCRRev** | CGGCTGAGGAAATTGAGAAG |
| **CRP2 (ANID_05960)** | **CRP2qPCRFwd** | TCGGCATTAACGCTCTTCAC |
|  | **CRP2qPCRRev** | ACCGATTCTCATACCGGAAC |
| **TEF1 (ANID_2063)** | **TEF1qPCRFwd** | GATTTTGGGCGATTTGAAGA |
|  | **TEF1qPCRRev** | GCGTGGAAGTACGGTTTTGT |
